# The efficacy of selected Ghanaian herbal antimalarials against laboratory strains and clinical isolates of *Plasmodium falciparum*

**DOI:** 10.1038/s41598-025-19429-1

**Published:** 2025-10-09

**Authors:** Silas N. Yeboah, Mina Ansong, Deborah Clotworthy, Priscilla E. Domie, Jersley D. Chirawurah, Collins K. Awiaga, Charles Mensah, Mona-Liza E. Sakyi, Edem Adika, Collins M. Morang’a, Gordon A. Awandare, Yaw Aniweh, Lucas N. Amenga-Etego

**Affiliations:** 1 West African Center for Cell Biology of Infectious Pathogens (WACCBIP), College of Basic and Applied Sciences, University of Ghana, Legon, Accra Ghana; 2 Department of Biochemistry, Cell and Molecular Biology, College of Basic and Applied Sciences, University of Ghana, Legon, Accra Ghana

**Keywords:** Antimicrobials, Parasitology, Pathogens

## Abstract

The recurrent emergence of drug-resistant *Plasmodium* parasites threatens malaria control and elimination strategies. In Ghana, there is a growing traditional medicine (TM) sector to support health delivery. In particular, the popularity of these alternative herbal malaria treatments is rapidly growing due to easy access, low cost, and perceived benefits. Some of these herbal malaria medicines are indicated to contain compounds that are in the same chemical classes as standard ACT partner drugs (e.g., quinolines). Thus, this study sought to evaluate the efficacy of selected antimalarial herbal drugs against *Plasmodium falciparum* parasites. In the study, twenty commercially available liquid formulations of antimalarial herbal medicines were obtained from drug stores and pharmacies within Madina, Accra, Ghana. The formulations were lyophilized and reconstituted into 50 mg/ml stock solutions. Following this, in vitro growth inhibition assays were used to assess the anti-plasmodial activity of these formulations against five laboratory strains and two clinical isolates of *P. falciparum*. The data from this study shows that of the 20 herbal products tested, 30.0% (*n* = 6) had potent activity across the six parasite strains screened with half-maximal inhibitory concentration (IC_50_) values of less than 50 ug/ml. In total, 3 of the 6 potent formulations were observed to exert their anti-plasmodial activity at the trophozoite stage whilst the rest showed varied activity in the various intraerythrocytic stages. These data highlight a couple of issues that have broad public health implications. (1) majority of herbal preparations tested showed poor anti-plasmodial activity, (2) the current study also highlights the need to strengthen the regulatory role in the efficacy testing and quality monitoring of TMs in Ghana.

**Supplementary Information:**

The online version contains supplementary material available at 10.1038/s41598-025-19429-1.

## Introduction

Malaria remains a global health threat despite numerous interventions through concerted efforts of various National Malaria Elimination Programs (NMEPs). In 2023, global malaria cases rose to an estimated 263 million (95% CI: 238–294 million), exceeding 2022 figures by 12 million^1^. The WHO African Region accounted for about 94% of global cases, with just five countries, Nigeria, DR Congo, Uganda, Ethiopia, and Mozambique contributing over half of the global disease burden. This setback in disease control and elimination is largely hampered by the rapid emergence of drug resistant *Plasmodium falciparum* parasites to front-line artemisinin-based combination therapies (ACTs). In Ghana, the front-line ACTs include Artesunate-Amodiaquine (AS-AQ), Dihydroartemisinin-Piperaquine (DHAP) and Artemether-Lumefantrine (AL). However, artemisinin resistance was reported in southeast Asia in 2008 and has more recently been confirmed in East Africa^2–4^.

Key among the factors that favor the development of resistance include the fitness cost of the mutation, the pharmacokinetic profile of the drug, and the pattern of drug use among others^5–7^. In such situations resistant parasites will be selected when exposed to subtherapeutic drug concentrations^8^. Thus, an important source of new resistance mutations are young children with little to no immunity with heavy infections who receive inadequate treatment due to poor drug quality, inaccurate treatment regimen (e.g. herbal preparations) or poor adherence^9^.

Over 80% of the rural population in Africa depends on traditional herbal remedies mainly because of access, low cost, and perceived benefits^10–12^. In Ghana, herbal medicine is an important strand of the health care delivery system^13^, and efforts are being made to integrate herbal medicine into mainstream healthcare^12^. In two districts of the Ashanti region of Ghana alone, a survey of medicinal plants and finished marketed products indicated for management of malaria reported 98 plants species and 22 finished marketed products with FDA approvals^14^. Undoubtedly, because Africa is endowed with a rich biodiversity, which has a wealth of medicinal plants traditionally used for treating various ailments, including malarial fever, the popularity of herbal medicines as an alternative to conventional orthodox medicines will continue to soar^15,16^.

While some herbal medicines have shown promising results in treating malaria, there is concern that lack of rigorous quality regulation and post licensure monitoring could contribute to the emergence and selection of resistance^17^. Despite the widespread use of herbal medicine indicated for malaria treatment, there is limited scientific evidence to support the dose regimen, efficacy, and safety of these herbal products as antimalarials. There is a growing concern that the concurrent use of herbal products and conventional drugs could contribute to the selection of resistant parasite strains, due to sub-therapeutic drug concentrations and poor pharmacokinetics that favors the survival of resistant parasites. Studies have also shown that the continuous exposure of malaria parasites to reduced concentrations of the antimalarial increases the chances of parasites developing *de-novo* mutations, which can rapidly spread when there is a fitness advantage in the presence of a particular front-line drug^18,19^.

Medicinal plants remain a major source of novel compounds in the drug discovery pipeline^20–22^. However, notwithstanding the existence of a large herbal medicine market in Ghana, there is paucity of data on the efficacy of these herbal products including preparations indicated for the treatment of malaria.

Therefore, the aim of this study is to investigate the efficacy of selected herbal products approved by FDA for the treatment of malaria. The study evaluated the efficacy of twenty (20) herbal products against five selected laboratory strains and two clinical isolates of *P. falciparum*. The killing rate and stage-specific activity of the most potent products were assessed using 3D7 parasites. The current data will throw more light on the importance of characterizing local herbal preparations used in the treatment of malaria.

## Methods

### Identification and selection of herbal products

The herbal products for this study were obtained from local herbal shops within the Madina community situated in Accra, Ghana. Only herbal drugs formulated for the treatment of malaria were collected. The herbal products were then lyophilized at the Center for Plant Medicine Research at Mampong (CPMR), Ghana (product weight ranged from 200 to 500 mg). The lyophilized products were then weighed and reconstituted into a stock concentration of 50 mg/mL (250 mg in 5 mL) using RPMI supplemented with 5% Dimethyl Sulfoxide (DMSO). The reconstituted stock products were subjected to a gentle agitation at 37 °C and left overnight. Stock solutions were then filter sterilized through a 0.22 μm Whatman filter and stored in aliquots at -80 °C for future use.

### Parasite culture

Ethical approval for this study was obtained from the College of Basic and Applied Sciences ethics committees of the University of Ghana, School of Biological Sciences (Ethics Reference Number: ECBAS 030/21–22). For this study, laboratory-adapted strains of *P. falciparum* (3D7, NF54, Dd2, W2mef and K1) as well as two clinical isolates (A11 and A377) were maintained in culture with uninfected human group O + erythrocytes using a slightly modified version of the Trager and Jensen method^23^. Parasite cultures were maintained in a T75 cm^2^ culture flask to a parasitemia of ≥ 5% ring-stage parasitemia in complete parasite media (RPMI 1640 supplemented with 10% AlbuMAX II, 25 mM HEPES, 12.5 µg/ml gentamycin, hypoxanthine, 100 µM L- glutamine) before setting up the growth inhibition assays. Synchronous ring-stage parasite cultures were obtained by subjecting the cultures to 5% D-sorbitol treatment^24^. The parasites were maintained for one intraerythrocytic cycle (48 h) after synchronization to enable them to recover from the stress before diluting them to the required parasitemia for the drug assays.

### In vitro growth inhibitory assay

To assess the potency of the collected herbal antimalarial drugs against the laboratory strains and clinical strains, a protocol like that described by^25^ with some slight modifications was used. Briefly, a 96-well tissue culture plate was filled with 50 µl of serially diluted herbal products from 20 mg/ml to 2 pg/ml in triplicate wells. The wells were then supplemented with 50 µl of infected RBCs at 2% hematocrit (hct) and 2% parasitemia. RPMI without drug was used as the negative control. The plates were then placed in a Modular incubating chamber and gassed for about 30 s and then incubated at 37 °C for 72 h. After the incubation period, 80 µl of 1X SYBR Green I ysis buffer (50 mM Tris-HCL, pH 7.5 supplemented with 15 mM EDTA, 0.5% w/v saponin, and 10% v/v Triton X-100 in PBS) was added to each well to lyse the cells. The plates were then incubated at 37 °C for about 30–60 min. Fluorescence readings were then obtained on a calibrated microplate reader (ThermoScientific Varioskan™ LUX, VLBL00D0) at 485 nm excitation and 520 nm emission.

### Stage-specificity assay

To assess the potency of the herbal drugs against the individual intraerythrocytic stages of *P. falciparum*, parasites were subjected to two consecutive sorbitol treatments (48 h apart). After the second treatment, parasites were split into three separate T75 cm^2^ culture flasks. For the first flask, 2 hours post-synchronization (hps) ring-stage parasites (parasitemia ≥ 5% with at least 95% synchrony) were incubated with serially plated drugs (20 mg/mL to 2 pg/ml) as described earlier. At time 26 hps, trophozoite stage parasites from the second flask with about 94% synchrony were incubated with serially plated drugs (20 mg/mL to 2 pg/ml). Lastly, at 38 hps, schizont stage parasites obtained were treated as the other two stages as discussed in this section.

### Killing rate assay

To evaluate the extent of the killing action of the herbal products, synchronized parasites (ring-stage parasitemia ≥ 12%; synchrony > 95%), were pulsed with 500 µg/mL of four of the most potent drugs (TFM, ZHM, MSM, HBQ) for 6 h. After initial incubation for 6 h, herbal products were washed 3X with incomplete RPMI. Parasites were then maintained in culture at 4% hct with daily replacement of drug-free media and supplementation with uninfected human O^+^ RBCs. Daily total parasitemia and individual-stage parasitemia were estimated to with Giemsa-stained thin smears.

### Data analysis

For each strain tested, the fluorescence readings obtained from the Microplate reader were processed using Microsoft Excel Software (Version 2108[Build 14334.20090]). Processed data was then imported into GraphPad Prism (Version 9.1.2), and a non-linear regression model fitted to the data to generate sigmoidal dose-response curve per drug and to estimate IC_50_ values with 95% confident intervals (CI). For this study, R-squared values greater than 0.9 were considered a good fit. The IC_50_ values are presented here as geometric mean IC_50_ + geometric mean 95% CI, from independent experiments conducted in triplicates. The Kruskal-Wallis method was used for nonparametric comparisons of IC_50_ values between herbal products. Parasite killing rate (i.e. reduction in percent parasitaemia) was examined using test of equality of proportions in with continuity correction in the open-source R statistical software (R Core Team (2023). _R: A Language and Environment for Statistical Computing_. R Foundation for Statistical Computing, Vienna, Austria. <https://www.R-project.org/>). The level of statistical significance was taken as a P value of < 0.05.

## Results

### Herbal products evaluated

**Fig. 1:**
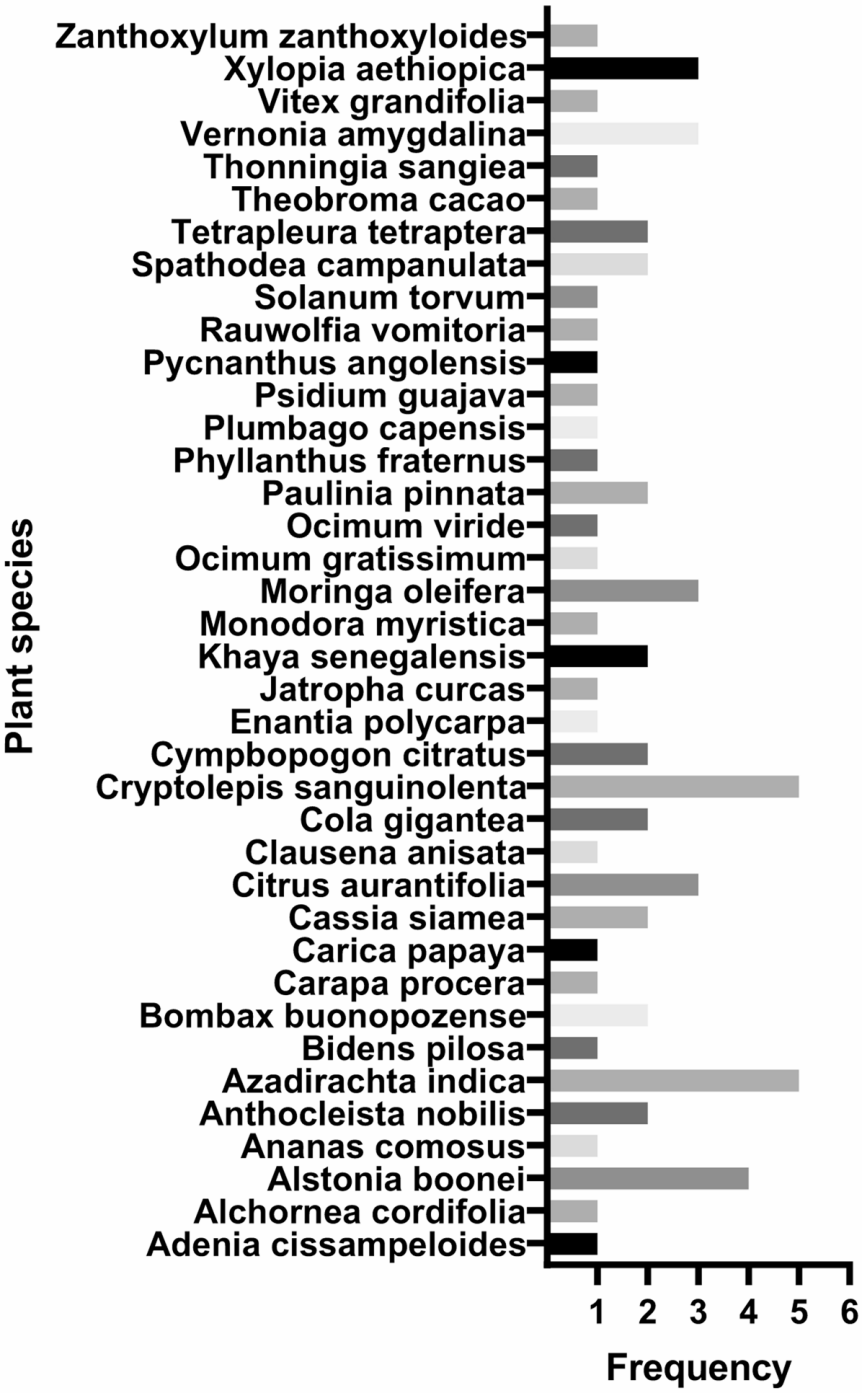
Common plant species used in the herbal products collected: The names of the thirteen different plant species with different frequencies were identified across the formulations collected.

In total, twenty (20) herbal products were collected for the study (Table 1). Thirty-eight (38) plant species were found to be the predominant constituents of the various products collected. Out of the 38 plant species, *Cryptolepis sanguinolenta* and *Azadirachta indica* were the most frequent, appearing in five of the herbal products (Fig. 1). *C. sanguinolenta* was present in PLM, MSM, TTM, HBQ and ADM whereas *A. indica* was present in THM, TFM, PLM, HBQ and GHM. *A. boonei* also appeared in four different herbal products.

**Table 1:** Summary of all collected drugs and their various ingredients.

Drug code names	Active ingredients
THM	*Ocimum viride*,* Azadirachta indica*,* Tetrapleura tetraptera*,* Theobroma cacao*,* Cymbopogon citratus*,* Moringa oleifera*
TFM	*Alstonia boonei*,* Azadirachta indica*
ADB	*Phyllanthus fraternus*,* Anthocleista nobilis*,* Vitex grandifolia*
LHM	*Khaya senegalensis; Rauwolfia vomitoria; Alstonia boonei; Pycnanthus angolensis*
LUM	*Moringa oleifera*,* Enantia polycarpa*,* Adenia cissampeloides*,* Plumbago capensis*,* Tetrapleura tetraptera*
ZHM	*Alchornea cordifolia*,* Zanthoxylum zanthoxyloides*,* Anthocleista nobilis*
PIM	*Moringa oleifera*,* Cymbopogon citratus*
MLM	*Cassia siamea*,* Citrus aurantifolia*
OHM	*Xylopia aethiopica*,* Paullinia pinnata*
GFM	*Vernonia amygdalina*,* Alstonia boonei*,* Ocimum gratissimum*
PLM	*Cryptolepis sanguinolenta*,* Azadirachta indica*
MSM	*Cryptolepis sanguinolenta*
PPD	*Carica papaya*,* Citrus aurantifolia*,* Jatropha curcas*,* Ananas comosus*,* Cassia siamea*
TTM	*Carapa procera*,* Cryptolepis sanguinolenta*
AHM	*Cola gigantea*,* Spathodea campanulata*,* Vernonia amygdalina*,* Solanum torvum*,* Bombax buonopozense*
HBQ	*Xylopia aethiopica*,* Cryptolepis sanguinolenta*,* Alstonia boonei*,* Monodora myristica*,* Azadirachta indica*
ADM	*Cryptolepis sanguinolenta*,* Thonningia sanguinea*,* Clausena anisata*
TIM	*Cola gigantea*,* Spathodea campanulata*,* Bombax buonopozense*,* Vernonia amygdalina*
GHM	*Khaya senegalensis*,* Azadirachta indica*
AGM	*Xylopia aethiopica*,* Citrus aurantifolia*,* Psidium guajava*,* Bidens pilosa*,* Paullinia pinnata*

### Growth Inhibition assay

**Fig. 2:**
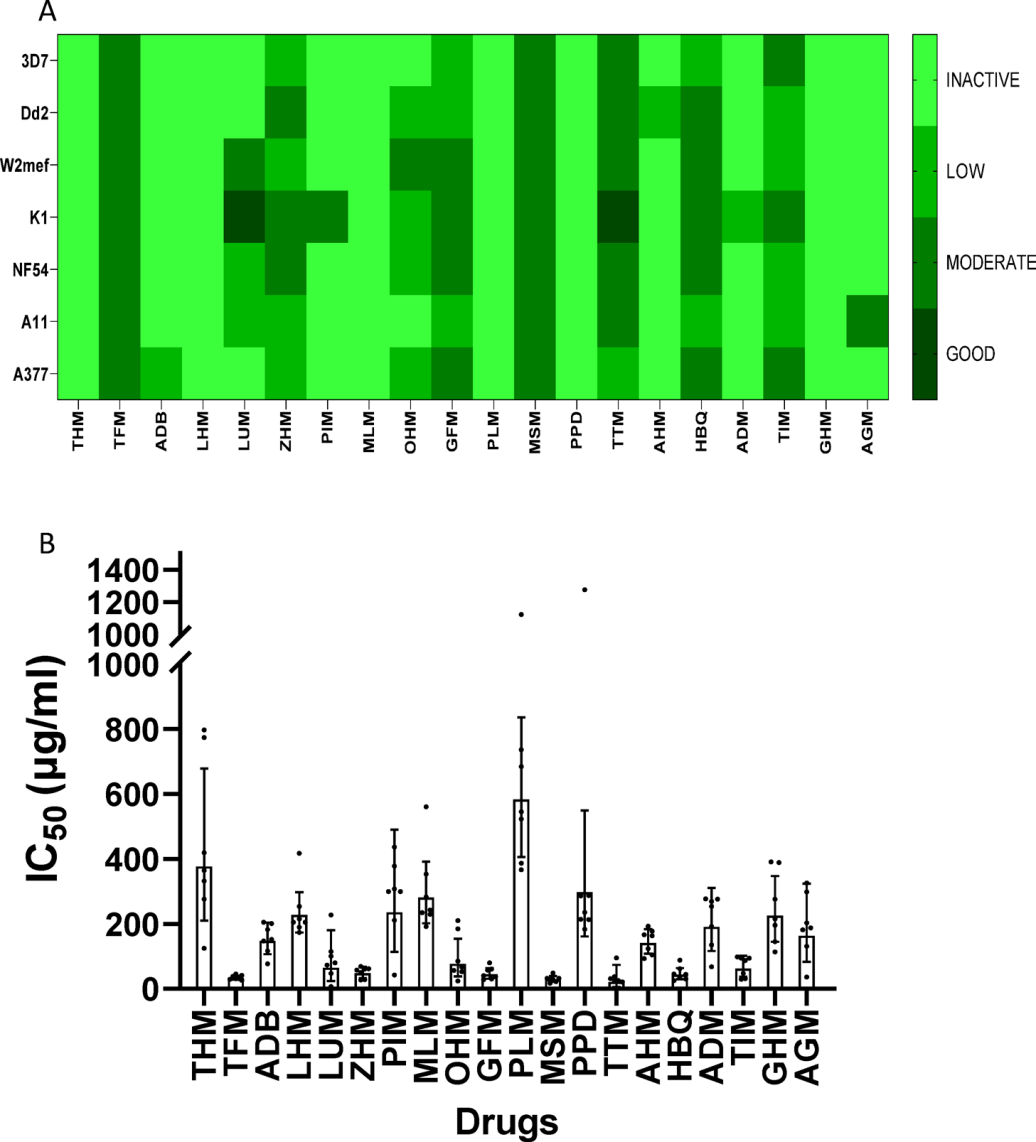
Activity of the different herbal products (**a**) Heatmap summary of the activities of herbal products against the different strains of *P. falciparum* strains tested (3D7, Dd2, W2mef, K1, NF54, A11 and A377). Color designations represent the activity of herbal products tested as good, moderate, low and inactive; gradient represents the IC_50_ values, with lighter shades indicating higher IC_50_ values (low drug potency) and darker shades indicating lower IC_50_ values (high drug potency). (**b**) A bar chart showing the geometric mean IC_50_ (µg/ml) of herbal products. Error bars represent the 95% CI with each data point representing a strain or isolate.

The twenty (20) herbal products collected were screened against five (5) laboratory strains (3D7, K1, Dd2, W2mef and NF54) and two (2) clinical isolates (A11 and A377) of *P. falciparum* parasites (Fig. 2A). The potency of the herbal products was classified as “good”, “low”, and “inactive” according to their half-maximal inhibitory concentrations (IC_50_) as follows: good activity IC_50_ ≤ 50 µg/ml, low activity 51 ≤ IC_50_ ≤ 100 µg/ml and inactive IC_50_ > 100 µg/ml. Out of the twenty herbal products tested, six had good activity against all screened parasites. These include TFM, ZHM, GFM, MSM, TTM and HBQ with geometric mean IC_50s_ of 33.71 µg/ml, 47.29 µg/ml, 44.63 µg/ml, 28.74 µg/ml, 20.42 µg/ml, and 43.12 µg/ml respectively (Fig. 2B). On the contrary, eleven (11) out of the twenty herbal products tested showed poor activity in the strains tested with IC_50_ values > 100 µg/ml (Fig. 2B).

### Stage-specific assay

**Fig. 3:**
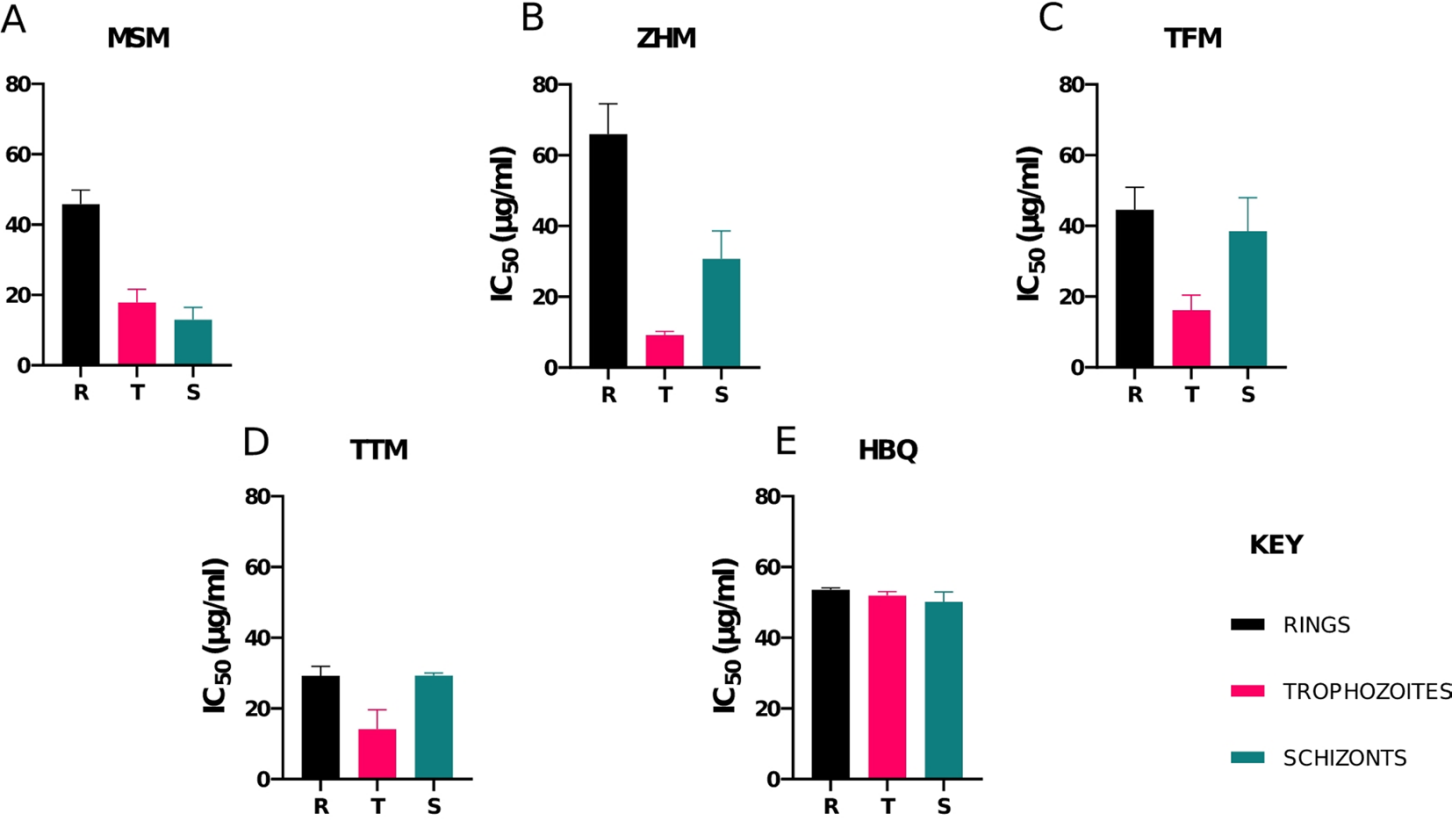
A bar chart showing stage-specific activity of the selected herbal products. (**A**) MSM (**B**) ZHM (**C**) TFM (**D**) TTM and (**E**) HBQ in (IC_50_ (ug/ml) against the asexual stages of 3D7 parasites. The stages range from Rings (R), Trophozoites (T) to Schizonts (S). Each bar represents the average IC_50_ value for each stage (*n* = 2). Error bars represent the standard deviation.

Five of the potent herbal products were screened against 3D7 strain of *P. falciparum* to assess their intraerythrocytic-stage (Rings, Trophozoites and Schizonts) activity (Fig. 3). MSM showed trophozoite and schizont stage activity with average IC_50s_ of 17.91 and 12.95 µg/ml respectively (Fig. 3A). ZHM showed prominent trophozoite stage activity with an average IC_50_ of 9.16 µg/ml (Fig. 3B). Both TFM and TTM showed trophozoite stage activity with average IC_50s_ of 16.19 µg/ml and 14.15 µg/ ml (Fig. 3C and D).

### Killing rate assay

**Fig. 4:**
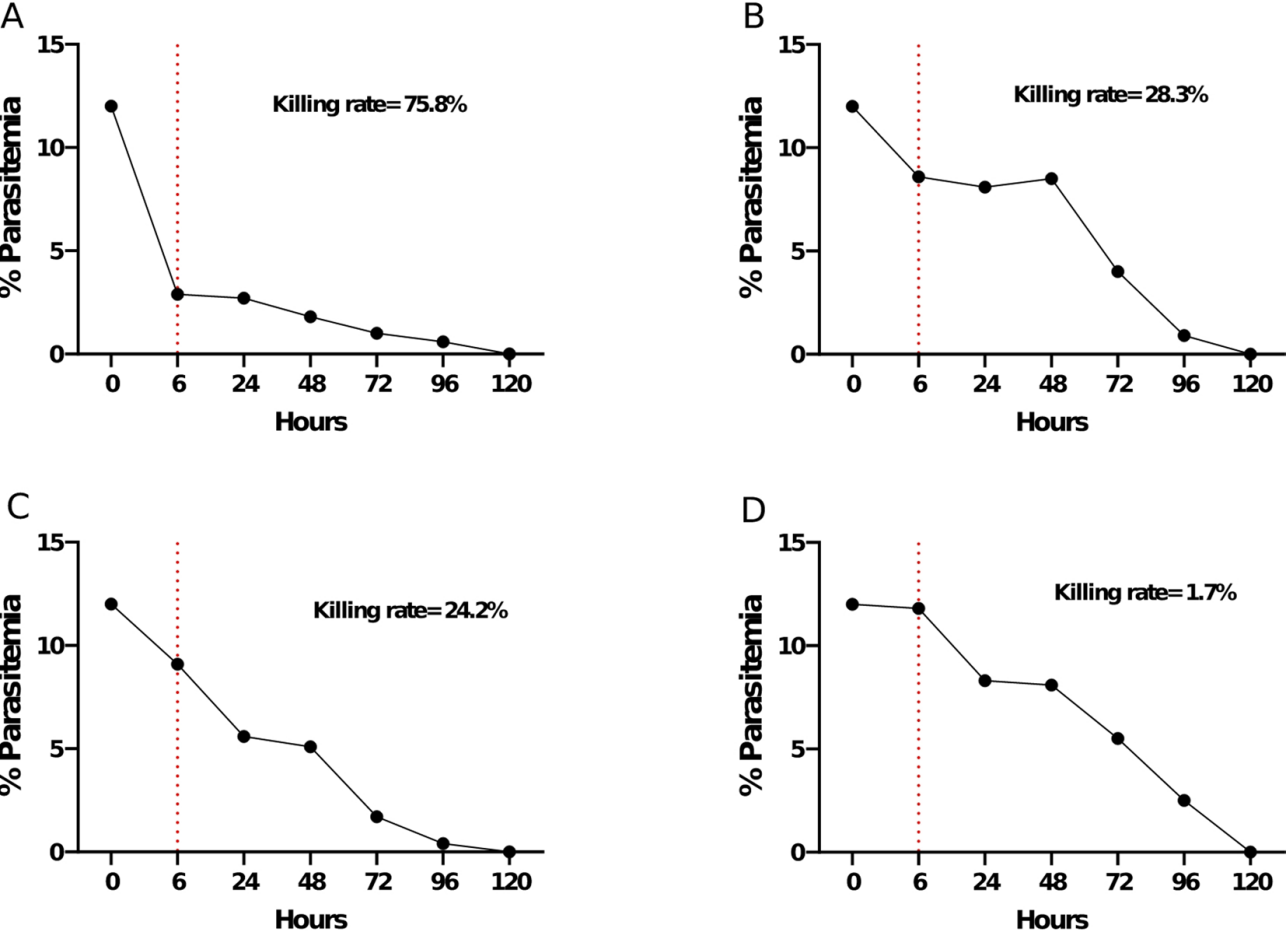
Killing rate of the selected active herbal products (**A**) MSM, (**B**) ZHM, (**C**) HBQ and (**D**) TFM after 72 h of continuous culture in drug-free media following an initial pulsing of parasites with 500 µg/ml of each drug for 6 h (red dotted line).

**Fig. 5:**
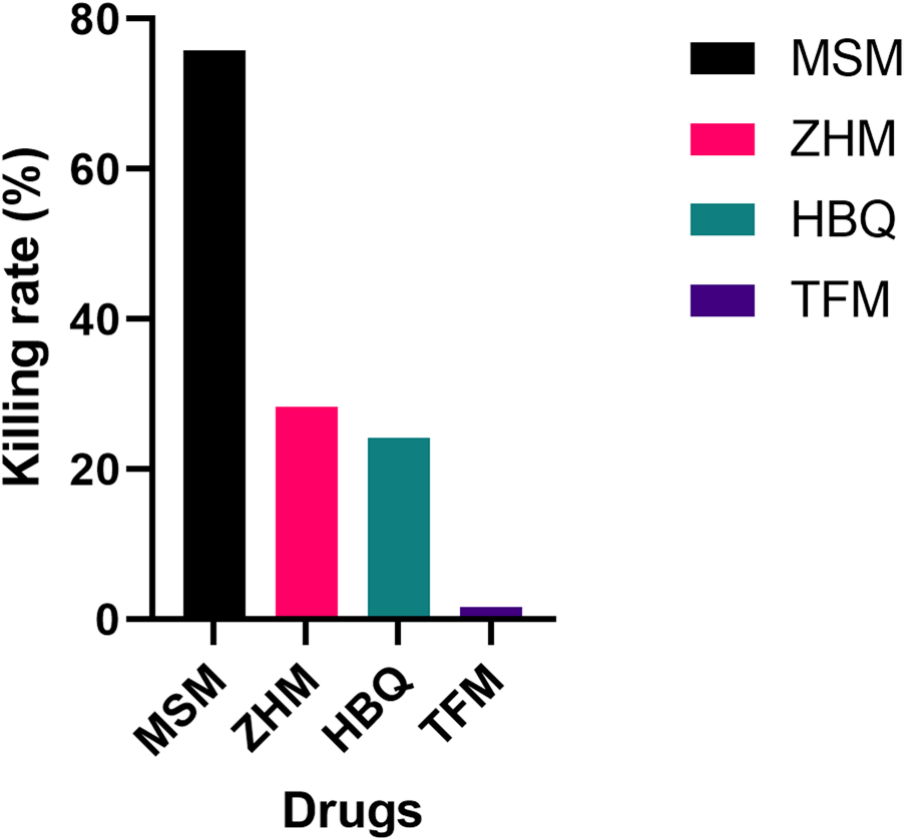
Summary of killing rate by MSM, ZHM, HBQ and TFM: The summary of the killing rate (%) (y-axis) for each of the herbal products (Drugs) (x-axis) estimated after 6 h exposure of the parasite. Black colour for MSM, magenda for ZHM, Green for HBQ and violet for TFM.

Parasites were exposed to 500 µg/ml of each drug for six (6) hours. After the six-hour exposure, a decrease in parasitemia was observed with all four herbal products (Fig. 4). MSM recorded a parasitemia of 2.9% corresponding to a killing rate of 75.8% marking a statistically significant killing rate within six hours of exposure compared to all other products (MSM vs. ZHM, 95%CI: [0.11–0.35], *p* = 0.0002, MSM vs. HBQ, 95%CI: [0.14–0.37], *p* = 3.3 e^− 05^, MSM vs. TFM, 95%CI: [0.39–0.56], *p* = 3.4 e^− 13^). (Fig. 4A). ZHM and HBQ recorded parasitemia of 8.6% and 9.0% corresponding to killing rates of 28.3% and 24.2% respectively (Fig. 4B and C). However, the rate difference between ZHM and HBQ was not statistically significant (95%CI: [-0.2-0.12], *p* = 0.727). TFM recorded the lowest killing rate at 1.7% with a parasitemia of 11.8% (Fig. 4D), which was significantly lower compared to MSM, ZHM and HBQ (Fig. 5). Despite the varied decline in parasitemia and killing rates after the six-hour exposure, there was a gradual decline in parasitemia for all drug-exposed cultures till no parasites were observed after 120 h of continuous culturing in drug-free media (Fig. 4). The killing rate was estimated as:

$$\:Killing\:Rate\:\left(\%\right)=\frac{Initial\:Parasitemia-New\:Parasitemia\:after\:6-hr\:exposure}{Initial\:Parasitemia}\times\:100$$

**Fig. 6:**
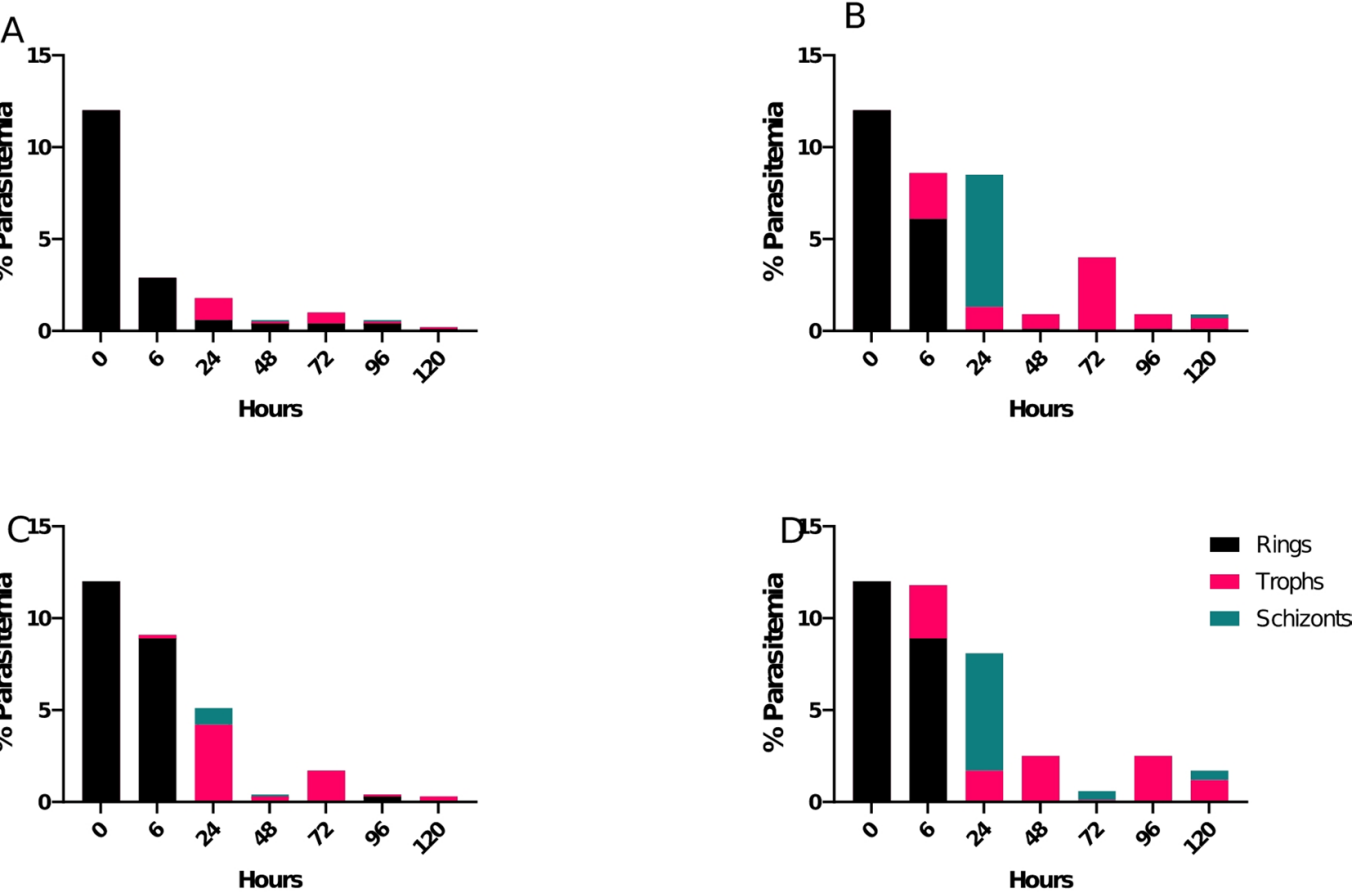
Time-point specific parasite stage progression. The percentage (%) parasitemia after the treatment with the selected herbal products: (**A**) MSM (**B**) ZHM (**C**) HBQ (**D**) TFM plotted against the incubation time up to 120 h of continuous culture in drug-free media following initial exposure of parasites to 500 µg/ml of each drug for 6 h. The stage specific transition is colour coded; black for rings, Pink for Trophozoites (trophs) and green for Schizonts.

The distribution of the parasites stages during the killing rate determination showed clearly that the different drugs tested act at different stages of the parasites growth. It was observed that MSM was very active at the ring stage of the development of the parasite (Fig. 6A). Its impact was very visible in the first cycle of parasite development. In the case of ZHM, the parasite progressed from rings to trophozoites and schizonts for the first cycle but were arrested and did not drive rings stage development in the second and third cycle. This indicates the drugs effect is in the transitioning from the first cycle to the second cycle suggesting a possible invasion inhibitory potential (Fig. 6B). A similar pattern was observed for HDQ. However, the transition to schizonts was impaired leaving more trophozoites persisting in the cultures (Fig. 6C). The TFM drug showed less effect on the different stages at the first cycle. The activity of the drug was evident in the second cycle where the effect is very pronounced (Fig. 6D).

## Discussion

The use of herbal remedies for treatment of illnesses has greatly increased worldwide in the recent times^26^. In Ghana, the integration of herbal medicines into mainstream healthcare as an alternative has been piloted by the Ghana Health Service, Ministry of Health. Despite the advantages of these treatment options, there exists a large traditional herbal market with little or no scientific data backing treatment efficacy claims in a largely unregulated herbal products ecosystem^25,27^. Therefore, this study evaluated the efficacy of 20 herbal products commonly used for treating malaria and assessed their efficacy and potential to contribute to the emergence of resistant *P. falciparum* parasites.

A total of 38 different medicinal plant products were found to be present in the herbal products collected. Out of the 20 herbal products, MSM was the only product that was formulated from a single plant, *Cryptolepis sanguinolenta*. *C. sanguinolenta* has been demonstrated to contain indoloquinoline alkaloids that possess potent antimalarial activity against various strains *P. falciparum*^28^. Seven of the herbal products were found to be formulated from two plant species and 12 were formulated from three or more plant species (Table 1). The variety of plants used in these products highlight the rich biodiversity of medicinal plants in Ghana^29^. Out of the 38 plants identified, *Azadirachta indica* (5), *Cryptolepis sanguinolenta* (5), and *Alstonia boonei* (4) were the most frequently used in the formulations. This observation is consistent with^30^ where *Azadirachta indica* (17), *Cryptolepis sanguinolenta* (15), and *Alstonia boonei* (12) were also found to be the most recurring plant species across 52 sampled herbal products in Ghana. These three plant species are well known for their antimalarial properties making them an ideal choice for manufacturers of herbal preparations^31–33^.

The antimalarial activity of these herbal plants has been linked to the presence of phytochemical compounds such as alkaloids^34^. Alkaloids have been shown to be involved in the inhibition of the detoxification of heme to hemozoin, leading to a gradual buildup of heme in the food vacuole of the parasite, ultimately killing the parasite^35^. Other plant compounds such as terpenoids, saponins, tannins among others have also been shown to be present in such medicinal plants. Specifically, the activity of *C. sanguinolenta* has been linked to the alkaloid, cryptolepine, *A. indica* has been shown to contain the terpenoid; azadirachtin whereas *A. boonei*, contains the alkaloids; alstonine and echtamine^36,37^.

In this study, TTM was found to be the most potent herbal product among the 20 tested followed by MSM with geometric mean IC_50_s of 20.4 µg/ml and 28.7 µg/ml respectively. In total, six products, in ascending order of activity; ZHM, GFM, HBQ, TFM, MSM, and TTM were found to have geometric mean IC_50_s corresponding to good activity (≤ 50 µg/ml) (Fig. 2B and Table S1). PLM and PPD were classified as having the lowest activities (> 100 µg/ml) among the products tested. However, further assays would be needed to validate this. This could be the case as certain plant species such as *A. indica* have been documented to have immunomodulatory properties^38^. Hence, the growth inhibition assay which measures direct parasite growth inhibition or killing might not fully reflect the activity of such drugs.

Furthermore, we examined the response of purified intraerythrocytic stages of *P. falciparum* parasites to the potent herbal products. HBQ showed relatively similar intraerythrocytic stage activity with average IC_50_ values ranging between 40 and 50 µg/ml. MSM demonstrated both trophozoite and schizont stage activity (Fig. 3A). This could be due to the presence of the phytochemical alkaloid groups. As mentioned earlier, one of the proposed mechanisms through which these alkaloid groups inhibit parasite growth is through the inhibition of heme detoxification^35^. At the S phase of the parasite’s cell cycle which coincides with the trophozoite stage, hemoglobin, in the RBC is digested for parasite growth and asexual replication^39^. However, preclinical development of indoloquinoline alkaloids has been hampered by their structural rigidity and associated cellular toxicity^40^. Chloroquine is a prominent quinoline drug that has been shown to inhibit the heme polymerase enzyme, has been reported to drive trophozoite-stage specific activity with selective toxicity to lysosomes of trophozoites^41^. A similar trend can be observed in these products, particularly HBQ, suggesting a broader mechanism of action that includes heme polymerization and degradation, double strand DNA intercalation and disruption of replication and transcription machinery^42^, like conventional drugs such as chloroquine. Such broad multi-stage activity is expected for herbal products as these usually contain a cocktail of compounds, which may either synergize to exert their individual effects across different parasite stages. This is interesting as some reports have suggested the intentional addition of chloroquine or similar derivatives to some herbal formulations for enhanced efficacy^43^. A similar parasite stage activity may confirm these observations or could suggest the presence of similar groups to chloroquine. This may be of concern as structural similarities may indicate the exposure of parasites to similar groups overtime ultimately leading to parasite resistance to these drugs. Indeed, in West Africa, notwithstanding the replacement of chloroquine monotherapy with artemisinin combination therapies over two decades ago, in most countries, chloroquine resistance alleles still exist at wide ranging frequencies^44–46^.

As a measure of drug activity, the ability of each herbal antimalarial drug to kill *Plasmodium falciparum* 3D7 parasites in vitro was assessed after drug exposure. MSM exhibited the most potent anti-plasmodial activity, as there was a sharp decline in parasitemia levels corresponding to a high killing rate. At a concentration of 500 µg/ml, MSM significantly reduced parasitemia levels compared to the other herbal drugs- ZHM, TFM, and HBQ (Fig. 5). Specifically, MSM achieved 75.8% inhibition, which was higher than the killing rates observed for the other drugs at the same concentration. As previously stated, structural similarity may underlie MSM’s enhanced efficacy. Cryptolepine (1,5-methyl-10 H-indolo[3,2-b] quinoline), shares the quinoline backbone of quinine and chloroquine and has been shown to disrupt the same biological processes in the malaria parasite as CQ, such as inhibiting heme detoxification within the parasite’s digestive vacuole^47^. By inhibiting this pathway, MSM likely induces rapid parasite death, leading to a rapid reduction in parasitemia. Thus, the widespread use of MSM in Ghana could potentially select existing parasite mutants that confer a fitness advantage, particularly if the pharmacokinetics of MSM results in subtherapeutic application.

## Conclusion

In this study, six commercial herbal products were identified to have potent anti-plasmodial activity. Our findings also demonstrate the stage-specific activity and varied killing rates of these herbal products with MSM having cross-stage activity and a rapid killing rate. Though 11 of the products tested fell within the inactive range, further assays would be needed to explore other potential pathways they could utilize to affect the malaria parasite. It is crucial to regulate the use of these products to curb the unintentional exposure of parasites to sub-therapeutic doses of compounds such as those contained in MSM and HBQ, which are like conventional antimalarials to prevent the selection of drug tolerant parasite strains with a fitness advantage which may lead to emergence of resistance to conventional antimalarial.

## Supplementary Information

Below is the link to the electronic supplementary material.

Supplementary Material 1

## Data Availability

The data available in this manuscript will be made available on request. Generally, all the original data have been used to generate the plots. These can be shared with any team, group, or individuals who need them. To assess the data in the manuscript, contact the corresponding author lamengaetego@ug.edu.gh.
