# Supplementary material for: The efficacy of selected Ghanaian herbal antimalarials against laboratory strains and clinical isolates of Plasmodium falciparum
Source: Sci Rep. 2025 Oct 9;15:35368. doi: 10.1038/s41598-025-19429-1 (PMC12511297; doi:10.1038/s41598-025-19429-1)
Supplement: Supplementary file 1 — Supplementary Material 1 [file 41598_2025_19429_MOESM1_ESM.docx]

The efficacy of selected Ghanaian herbal antimalarials against laboratory strains and clinical isolates of *Plasmodium falciparum*

Silas N. Yeboah^1^, Mina Ansong^1^, Deborah Clotworthy^1^, Priscilla E. Domie^1^, Jersley D. Chirawurah^1^, Collins K. Awiaga^1^, Charles Mensah^1^, Mona-Liza E. Sakyi^1^, Edem Adika^1^, Samirah Saiid^1^, Collins Morang’a^1^, Gordon A. Awandare^1,2^, Yaw Aniweh^1*^ and Lucas N. Amenga-Etego^1*^

^1^West African Center for Cell Biology of Infectious Pathogens (WACCBIP), College of Basic and Applied Sciences, University of Ghana, Legon, Accra

^2^Department of Biochemistry, Cell and Molecular Biology, College of Basic and Applied Sciences, University of Ghana, Legon, Accra

*For correspondence: [lamengaetego@ug.edu.gh](mailto:lamengaetego@ug.edu.gh) and [yaniweh@ug.edu.gh](mailto:yaniweh@ug.edu.gh)

Supplementary tables

**Supplementary table S1.** Geometric mean IC50s of the herbal products and Artesunate (AS) control across all strains tested

| **Products** | **IC50 (µg/ml)**  **(95% CI)** | **Products** | **IC50 (µg/ml)**  **(95% CI)** | **Products** | **IC50 (µg/ml)**  **(95% CI)** |
| --- | --- | --- | --- | --- | --- |
| THM | 377  (209 – 678) | MLM | 281  (202 – 392) | AHM | 141  (109 – 183) |
| TFM | 33.7  (28.0 – 40.5) | OHM | 76.7  (38.3 – 154) | HBQ | 43.1  (29.0 – 64.0) |
| ADB | 147  (107 – 203) | GFM | 44.6  (31.1 – 63.9) | ADM | 190  (116 – 311) |
| LHM | 227  (174 – 298) | PLM | 583  (406 – 835) | TIM | 61.2  (36.9 – 101) |
| LUM | 65  (23.5 – 180) | MSM | 28.7  (21.2 – 39.0) | GHM | 225  (145 -347) |
| ZHM | 47.3  (33 – 67.7) | PPD | 298  (161 - 549) | AGM | 163  (82.5 – 324) |
| PIM | 235  (113 – 490) | TTM | 20.4  (5.69 – 73.3) | AS_ctrl | 1.08  (0.256-4.52) |
